# A Conditioning Lesion Provides Selective Protection in a Rat Model of Amyotrophic Lateral Sclerosis

**DOI:** 10.1371/journal.pone.0007357

**Published:** 2009-10-06

**Authors:** Colin K. Franz, Eric T. Quach, Christina A. Krudy, Thais Federici, Michele A. Kliem, Brooke R. Snyder, Bethwel Raore, Nicholas M. Boulis

**Affiliations:** 1 Department of Neurosurgery, Emory University, Atlanta, Georgia, United States of America; 2 Department of Neurology, Emory University, Atlanta, Georgia, United States of America; University of Giessen, Germany

## Abstract

**Background:**

Amyotrophic Lateral Sclerosis (ALS) is neurodegenerative disease characterized by muscle weakness and atrophy due to progressive motoneuron loss. The death of motoneuron is preceded by the failure of neuromuscular junctions (NMJs) and axonal retraction. Thus, to develop an effective ALS therapy you must simultaneously preserve motoneuron somas, motor axons and NMJs. A conditioning lesion has the potential to accomplish this since it has been shown to enhance neuronal survival and recovery from trauma in a variety of contexts.

**Methodology/Principal Findings:**

To test the effects of a conditioning lesion in a model of familial ALS we administered a tibial nerve crush injury to presymptomatic fALS^G93A^ rats. We examined its effects on motor function, motoneuron somas, motor axons, and NMJs. Our experiments revealed a novel paradigm for the conditioning lesion effect. Specifically we found that the motor functional decline in fALS^G93A^ rats that received a conditioning lesion was delayed and less severe. These improvements in motor function corresponded to greater motoneuron survival, reduced motor axonopathy, and enhanced NMJ maintenance at disease end-stage. Furthermore, the increased NMJ maintenance was selective for muscle compartments innervated by the most resilient (slow) motoneuron subtypes, but was absent in muscle compartments innervated by the most vulnerable (fast fatigable) motoneuron subtypes.

**Conclusions/Significance:**

These findings support the development of strategies aimed at mimicking the conditioning lesion effect to treat ALS as well as underlined the importance of considering the heterogeneity of motoneuron sub-types when evaluating prospective ALS therapeutics.

## Introduction

Amyotrophic lateral sclerosis (ALS) is a progressive neurodegenerative disease characterized by the loss of motoneurons. The majority of ALS cases are sporadic in origin (∼90%), and the remaining 10% of cases are the inherited form, commonly referred to as familial (f)ALS [1]. For more than a decade Riluzole has remained the only medication proven to slow, albeit very modestly, the progression of ALS. This lack of success in ALS therapeutic development has not been due to lack of effort. Rather it seems to be reflective of our incomplete understanding of disease mechanisms. For example, only recently has it become widely accepted that maintaining motor axons and neuromuscular junctions (NMJs) are distinct from, but no less important than preventing motoneuron cell death in ALS [2]. Thus, developing therapies aimed at preserving both motoneurons and their connections with muscle should be prioritized.

Conditioning lesions have been shown to augment neuronal survival and recovery following a subsequent injury. In the PNS, conditioning lesions have been shown to enhance regeneration rate [3], [4] and the extent of axon collateral sprouting [5], [6]. If dorsal root crush injury is preceded by a peripheral nerve axotomy the ability for regenerating axons of the central process to overcome inhibitory growth cues is increased [7], [8], [9]. Additionally, sub-lethal insults to basal forebrain neurons have been shown to confer neuroprotection [10]. Therefore the present study set out to study the effects of a conditioning lesion applied to presymptomatic fALS^G93A^ rats on the preservation of motor function, motoneurons, motor axons and NMJs.

## Results

### Conditioning lesion focally reduced motor functional decline

**Figure 1:**
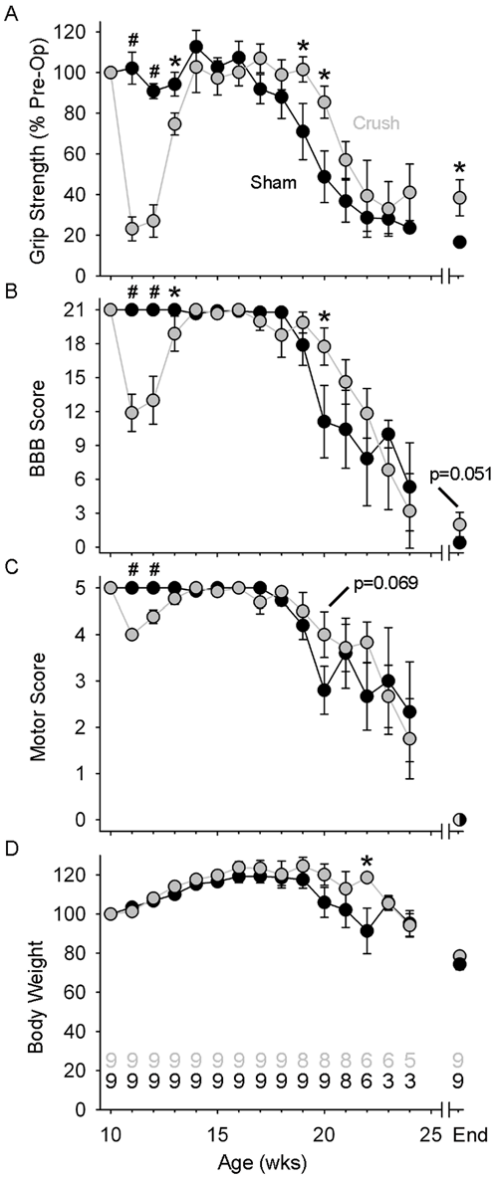
Conditioning lesion reduces the decline of motor function. (A) The mean (± SE) hindlimb grip strength for Crush (grey circles) and Sham (black circles). (B) The mean (± SE) BBB locomotor score relative for Crush and Sham. (C) The mean (± SE) ALS motor score for Crush and Sham. (D) The mean (± SE) body weight for Crush and Sham. The number of animal assessed in each group over time is indicated at the bottom of graph D. #, *P*<0.01; *, *P*<0.05.

To determine the effects of a conditioning lesion on disease progression in fALS^G93A^ rats we crushed the tibial nerve at age 10 weeks (wk). We considered this a conditioning lesion because it was administered prior to the onset of both motoneuron loss occurs (13–16 wk) and muscle weakness (16–20 wk) [11]. Following tibial nerve crush the function of the ankle extensor muscles was severely compromised and behavioral recovery took 3–4 wk (Figure 1A–C). The relatively large decreases in hindlimb grip strength (GS; −76.8±5.9%) and BBB locomotor rating (−43.3±8.1%) 1 wk after nerve injury indicated these tests were highly sensitive to tibial nerve function. In contrast, nerve injury had lesser or no effects on ALS motor score (−20.0±0.0%) and body weight (+1.3±1.1%) respectively, which was consistent with the more holistic nature of these tests. From ages 15–18 wk, all behavioral tests produced stable results in both groups. At 19 wk, hindlimb GS was significantly reduced in Sham (75.7±13.3%) versus Crush (101.5±6.2%; p = 0.028). At 20 wk, both hindlimb GS (55.0±6.2% vs. 85.4±8.0%; p = 0.011) and BBB score (12.1±3.0 vs. 17.8±1.7; p = 0.013) were decreased in Sham versus Crush. In contrast, the ALS motor score, which provides a more holistic view of motor function [11], did not vary significantly between groups at any time beyond age 12 wk. From age 20 wk onward all behavioral tests revealed a progressive decline in both groups. Body weight was similar between groups except at age 22 wk (p = 0.026), but this difference was likely an anomaly caused by the coincidental death of 3 Sham group animals between ages 22–23 wk (Figure 1D). In support of this interpretation, end-stage body weight for Crush and Sham were not statistically different (74.4±2.8% vs. 78.5±2.3%; p = 0.196). However, when motor function was compared at end-stage the Crush group outperformed Sham in hindlimb GS (38.4±8.8% vs. 16.6±1.9%; p = 0.012) and BBB (2.0±1.1 vs. 0.36±0.38; p = 0.051). Finally, at end-stage the ability to move the ankle joint extensively remained more frequently in Crush (5/9 or 56%) than in Sham (1/9 or 11%) rats.

### Disease onset, duration and survival were not altered

**Figure 2:**
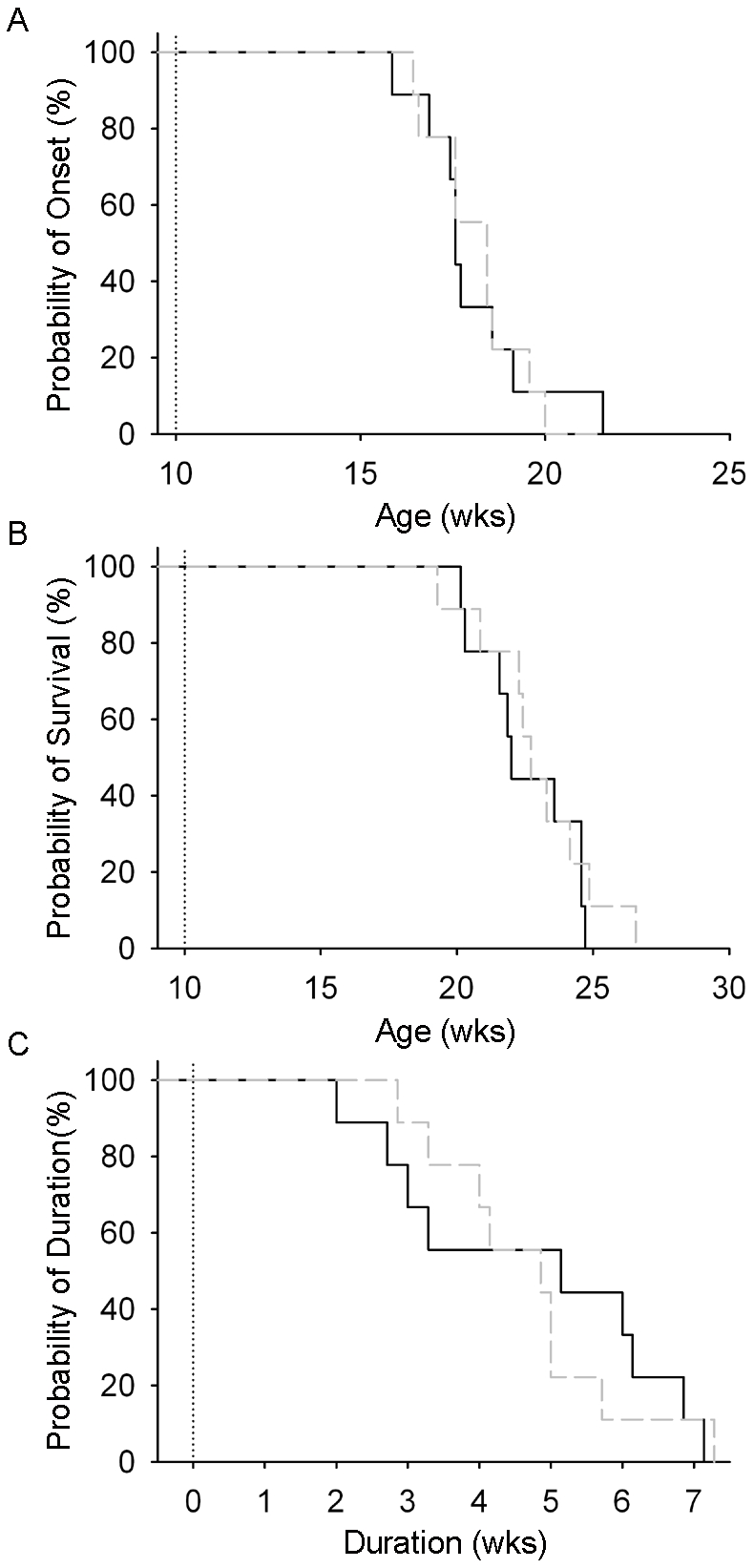
Conditioning lesion does not alter disease onset, survival or duration. (A) The onset of disease was similar for Crush (grey broken line) and Sham (black line) groups. (B) The age of death was not changed by the conditioning lesion. (C) The duration of the disease did not differ between groups.

The behavioral benefits of a conditioning lesion in fALS^G93A^ rats were focal (Figure 1A–B) rather than holistic (Figure 1C–D). Whether the focal preservation of ankle extensor function could alter disease progression seemed doubtful. To confirm this we compared disease onset, duration and survival between Crush (n = 9) and Sham (n = 9). Disease onset was defined objectively as the point where maximum body weight was reached and did not vary with Sham (17.9±0.5 wk) or Crush (18.3±0.5 wk). Furthermore, neither disease duration (4.7±0.7 wk vs. 4.7±0.5 wk) nor survival (22.6±0.7 wk vs. 22.9±0.8 wk) differed between Sham and Crush respectively (Figure 2).

### Conditioning lesion decreased motoneuron loss

**Figure 3:**
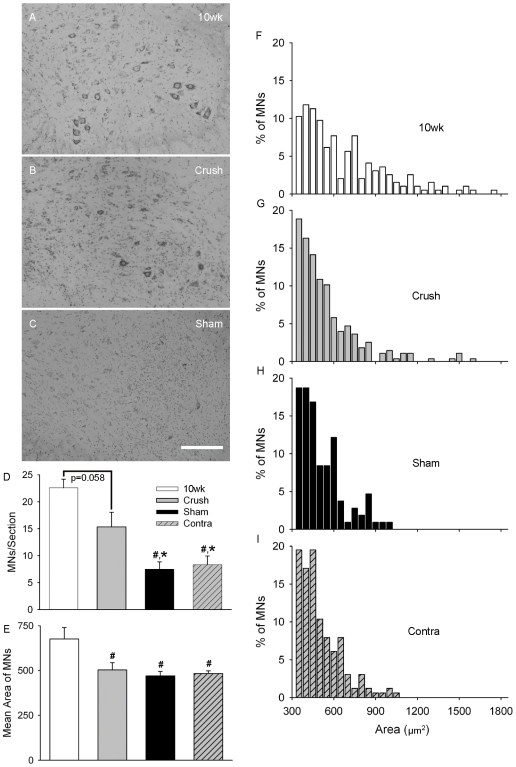
Conditioning lesion protects motor neurons at end-stage. (A–C) Representative examples of Nissl stained ventral horns from the L4–6 spinal cord of 10 wk un-operated, Crush, and Sham. (D) The mean (± SE) density of motor neurons per section compared between 10 wk, Crush, Sham and the contralateral side of Crush (Contra). (E) The mean (± SE) area of motor neuron somas shown for each group. (F–I) Histograms show the distribution of motor neuron areas for each group. Scale bar represents 250 µm. #, *P*<0.01; *, *P*<0.05.

**Figure 4 pone-0007357-g004:**
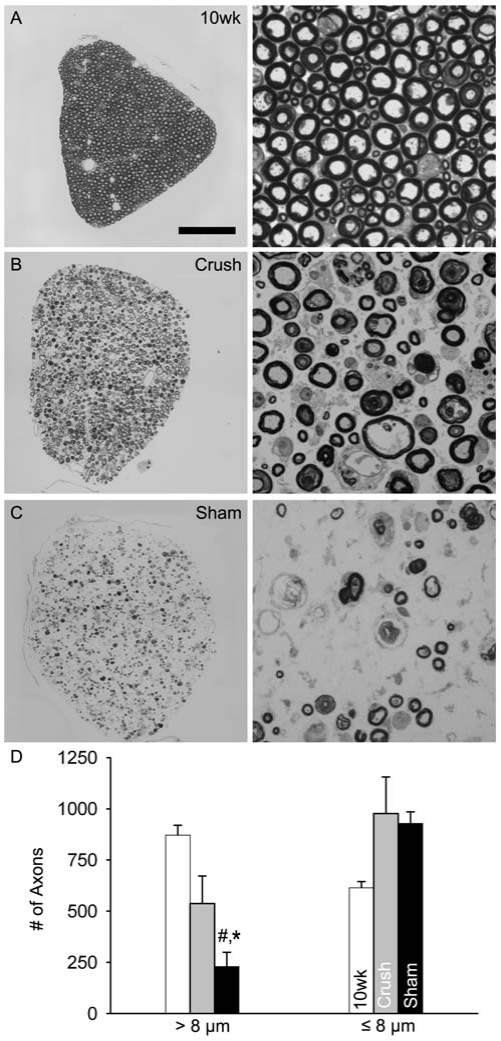
Conditioning lesion preserves ventral root axons at end-stage. (A–C) Photomicrographs of L4 VRs stained with toluidine blue and taken at either 20 or 100X. (D) The mean (± SE) number of large (black bars; >8 µm) and small (white bars; ≤8 µm) myelinated axons. Scale bar represents 150 µm (20X) or 25 µm (100X). #, *P*<0.01; *, *P*<0.05.

In fALS^G93A^ rodents, there are conflicting reports as to whether a previous nerve injury increases [12], [13], [14] or decreases [15] the vulnerability of motoneurons. We explored this issue by comparing the density of L4–6 motoneurons at end-stage of Crush and Sham fALS^G93A^ rats. Relative to 10 wk (pre-operative age), motoneurons at end-stage in Crush seem better preserved than in Sham (Figure 3A–C). The motoneuron density for 10 wk controls (22.6±1.6) had a tendency to be greater than end-stage Crush (15.3±2.7; p = 0.058). However, both 10 wk (p<0.01) and Crush (p<0.05) had significantly greater density than either Sham (7.5±1.3) or the unoperated side (i.e. Contra) of Crush (8.3±1.6). Consistent with the significant protection of motoneurons, we saw clear differences in the number of myelinated axons in 10 wk (n = 4), Crush (n = 4) and Sham (n = 4) L4 ventral roots (VRs; Figure 4A–C).

It is recognized that larger motoneurons are more susceptible to atrophy and/or death in ALS [16], [17], [18]. So we considered the affects of axotomy on the size of the remaining motoneurons (Figure 3E). We found 10 wk (676.3±63.6 µm^2^) motoneurons were significantly larger (p<0.01) as compare to Crush (503.6±40.5 µm^2^), Sham (470.5±24.1 µm^2^) and Contra Crush (483.1±15.2 µm^2^). Interestingly, histogram analysis of motoneuron areas revealed that while sizes for Crush (n = 276 cells/6 rats) were shifted towards smaller values relative to 10 wk (n = 195 cells/4 rats), when compared to either Sham (n = 107 cells/6 rats) or Contra Crush (n = 164 cells/6 rats) noticeably more large cells (>1000 µm) remained in Crush (Figure 3F–I). Similarly, quantification of L4 VRs showed that the number of large diameter (>8 µm) axons was significantly reduced for Sham (229.3±70.3) as compared to Crush (537.7±133.5; p<0.05) and 10 wk (871.5±47.6; p<0.01), but there was no difference in the number of small diameter (<8 µm) axons between groups (Figure 4D). Taken together this indicates that Crush treatment prevents the loss and/or atrophy of at least some larger motoneurons.

### Conditioning lesion selectively preserved resilient NMJs

**Figure 5:**
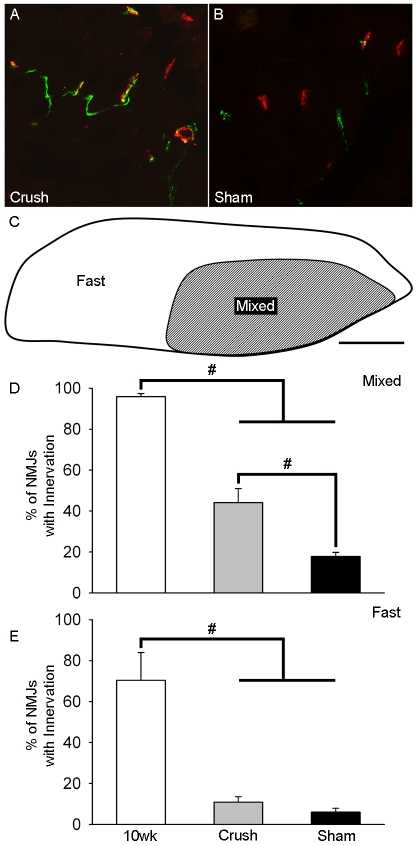
Conditioning lesion selectively preserves NMJs at end-stage. (A–B) Motor endplates were stained with α-bungarotoxin (red); axons were stained with antibodies for synaptophysin and class III β-tubulin (green). (C) A diagram depicting MG muscle compartments containing mixed (fast & slow) or only fast muscle fibers. (D–E) The mean (±SE) percentage of innervated NMJs was quantified for the mixed and fast muscle compartments of the MG muscle from 10 wk, Crush, and Sham. Scale bar represents 50 µm (A, B) or 1 mm (C). #, *P*<0.01; *, *P*<0.05.

The death of motoneurons in ALS is preceded by the loss of NMJs and axonopathy. Recently it has been shown that NMJs are more severely compromised in fast versus slow muscle fibers of fALS^G93A^ mice [19], [20]. The extent to which a conditioning lesion affects innervation at end-stage in distinct compartments of the fALS^G93A^ rat MG containing either mixed (∼50% fast/50% slow) or only fast fibers was examined with immunohistochemistry (Figure 5A–C). In the mixed region, the percentage of innervated NMJs was greater for Crush (44.1±6.9%, n = 532 NMJs/6 rats) than Sham (17.9±1.9%, n = 618 NMJs/6 rats, p<0.01), but compared to 10 wk (95.8±1.6%, n = 366 NMJs/4 rats, p<0.01) both were less (Figure 5D). In the fast region, the percentage of innervated NMJs was not different between Crush (10.9±2.7%, n = 182 NMJs/6 rats) and Sham (6.0±1.9%, n = 217 NMJs/6 rats; p = 0.168), but compared to 10 wk (70.4±13.6%, n = 98 NMJs/4 rats, p<0.01) both were less (Figure 5E). The percentage of innervated NMJs was not different between the fast and slow regions at 10 wk (p = 0.160), but did differ at end-stage for both Crush (p<0.01) and Sham (p<0.01).

## Discussion

We have shown that a conditioning lesion in fALS^G93A^ rats reduced motor functional decline, motoneuron loss, axonopathy, and muscle denervation in a focal manner. In addition, the conditioning lesion-induced enhancement of protection appeared to be exclusive for already ALS-resistant motoneuron subtypes. This represents a significant advance from previous studies of nerve injuries in fALS^G93A^ rodents, which either didn't allow muscle reinnervation to occur [13], [14], [15] and/or only studied their effects at intermediate stages of disease progression [12], [13], [14], [21]. Overall, our findings describe a novel paradigm for the conditioning lesion effect as well as imply that strategies aimed at mimicking this effect could be effective ALS therapies.

### Nerve Injuries and fALS

It seems counter-intuitive that a nerve injury could improve the maintenance of motor function and neuroprotection in a model of fALS. Axotomy itself can threaten the survival of motoneurons depending on age, motoneuron type, and nature of the lesion [22]. Complete recovery of function after peripheral nerve injury is rarely achieved [23]. In addition, the processes of axon regeneration and synaptogenesis place metabolic burden on already vulnerable cells. However, past research exploring the effects of nerve injury in fALS^G93A^ rodents have reported both protective [15] and detrimental [12], [13], [14] effects on motoneuron survival. For example, following sciatic nerve injury Kong and Xu found 33% more VR axons at end-stage [15], but later studies that examined sciatic [12] or facial[13], [14] nerve injury found 5–47% fewer motoneurons at disease onset.

The reason for these discrepancies is likely multifactorial. First, the age of injury must be considered because ALS involves progressive neurodegeneration. The aforementioned studies, plus our own administered nerve injury to fALS^G93A^ rodents ranging from ages 6–10 wk, which corresponds to a disease phase just after the onset of axonopathy but prior to the loss of motoneurons or physical symptom onset [11], [18]. Thus, these relatively minor variations in age of injury seem insufficient to account for these widely discrepant results. Second, due to their greater dependence on target-derived trophic support, adult cranial motoneurons are much more susceptible to axotomy-induced cell death than adult spinal motoneurons [22]. Hence, survival of adult facial and sciatic motoneurons following axotomy should not be directly equated [13]. Third, the nature of the nerve injury (e.g. ligation vs. crush) either prevented or allowed muscle reinnervation. As expected, motor function was permanently impaired when muscle reinnervation was prevented by nerve ligation [15]. In contrast, muscle was more fatigue-resistant [12] and motor function was better maintained (Figure 1) when muscle reinnervation was allowed after nerve crush. Finally, the effects of axotomy were assessed at different phases of the disease. At disease onset, the survival of axotomized motoneurons was diminished [12], but at end-stage, survival of axotomized motoneurons was enhanced [15] (Figure 3). Therefore it appears that axotomy has opposing effects on motoneuron survival depending on what disease phase is used as the experimental end point.

To better account for these opposing effects we explored the heterogeneity of motoneuron loss in ALS [24]. Although ALS is characterized by the widespread loss of motoneurons, not all are afflicted equally. For example, evidence from both ALS patients and animal models suggest that motoneurons innervating the greatest numbers of muscle fibers are compromised earliest [25], [26], [27]. It has also been reported that motoneurons with the largest somas and axons are more likely to be lost and/or become atrophic [16], [17], [18]. These observations are congruent, since bigger motoneurons generally innervate more muscle fibers [28], and support the popular hypothesis that motoneurons are specifically lost in ALS due to the burden of maintaining large somas, long axons, and multiple synaptic connections with muscle fibers. Elegant studies by Kong and Xu provided strong experimental evidence for this hypothesis and suggested that a size threshold might determine motoneuron survival. Specifically, they found that chronic axotomy caused axonal atrophy in wild-type mice, protected VR axons in fALS^G93A^ mice, and increased the proportion of small diameter axons in fALS^G93A^ mice [15], [17]. However, evidence against a direct relationship between smaller size and ALS-resistance includes the absence of protection when axon caliber was genetically reduced in fALS^G37R^ mice [29], and our findings that the axotomy-induced protection in fALS mice was not exclusive to motoneurons with small somas (Figure 3) and axons (Figure 4).

Since motoneuron size does not fully explain the effects of axotomy we considered motoneurons in terms of function. Corresponding to the muscle fiber type it exclusively innervates (Type I or II) a motoneuron can functionally defined as slow (S) or fast (F) respectively. F motoneurons can be further described as fatigable (F_ff_; Type IIb), intermediate (F_fi_; Type IIx), and fatigue-resistant (F_fr_; Type IIa). Along with well described differences in contraction speed, strength and fatigability of their muscle fibers[23], these motoneuron subtypes differ in their vulnerability to degeneration in fALS^G93A^ mice [19], [20], [21], [26] and rats (Figure 5). The most susceptible are F motoneurons (F_ff_>F_fi,fr_), which withdraw much of their innervation pre-symptomatically, and the most resilient are S motoneurons, which do not begin to display signs of axon degeneration until approximately disease onset [21]. Not only is this consistent with the hypothesis that bigger motoneurons are more susceptible in ALS, since F motoneurons are generally larger and innervate more muscle fibers then S motoneurons [28], but it may also accounts for the absence of a simple relationship between size and vulnerability [29] (Figures 3 and 4), since the range of sizes for S and F motoneurons are known to overlap considerably [30]. Furthermore, strong evidence exists that the axotomy-induced protection in fALS^G93A^ rodents was selective for S motoneurons because reinnervated muscle became more fatigue-resistant [12], had greater oxidative capacity [12], and maintained more NMJs exclusively in sub-compartments with S innervation (Figure 5). Although far from conclusive, there is also evidence to suggest that axotomy may have the opposite effect on the most vulnerable (i.e. F_ff_) motoneurons. For example, when the sciatic nerve of fALS^G93A^ mice was crushed at age 5.5wk (i.e. >1.5 wk prior to the earliest disease-related denervation), reinnervation deficits were found 1 wk later selectively in muscle compartments that had F_ff_ innervation [21]. However, it is not known whether this observation represented an axotomy-induced acceleration of F_ff_ motoneuron degeneration and/or impairment of F_ff_ axon regeneration.

### Making medicine from mechanism

While understanding the mechanisms underlying the selective loss of motoneurons remains one of the greatest challenges in ALS research, exploiting the known mechanisms that confer resiliency to specific motoneuron subtypes provides a great opportunity to develop new therapies. For reasons that are poorly understood, functional subtypes of motoneurons have distinct vulnerabilities in ALS [24]. Greater resiliency is found in motoneurons that have smaller axons, lesser innervation ratios, and synapse with muscle fibers that are slow contracting and more oxidative [19], [20], [21], [26], [27]. Pre-symptomatic nerve injury in fALS^G93A^ rodents (i.e. conditioning lesion) selectively increases the resiliency of motoneurons possessing some or all of these characteristics [12], [15] (Figures 3–5). Taken together this highlights the relevance to ALS of exploring: (i) the factors underlying the growth and survival of specific motoneuron types; and (ii) strategies to transform motoneurons from vulnerable (e.g. F_ff_) to resilient (e.g. S) phenotype.

Regarding the first point, peripheral nerve axotomy is known to cause the upregulation of various neurotrophic factor (NFs) and their receptors [31]. NF-based therapies have been among the most effective therapeutic approaches tested in preclinical models of ALS [32], and could conceivably underlie the effects seen in the present study. It is well established that different classes of neurons are responsive to different NFs or combinations of NFs. Even within classes of neurons, such as motoneurons, there exists considerably heterogeneity in responsiveness to specific NFs [33]. For example, the application of Neurotrophin-3 (NT-3) to regenerating axons appears to selectively augment the regeneration of F_ff_ motoneurons [34], [35] because of their relatively high expression of its corresponding tyrosine kinase receptor, trkC [36], whereas NT-4 appears to selectively augment the regeneration of S motoneurons [37] probably because they express relatively more of the trkB receptor [38]. However, the extensive intermingling of F and S motoneurons in the spinal cord, combined with absence of distinguishing molecular markers, have hampered efforts to explore the molecular heterogeneity of F and S motoneurons more definitively. Future studies directed towards increasing our knowledge of the variability between motoneuron subtypes might be leveraged to optimize the impact of growth factors, which, while promising [39], have so far failed to translate into ALS therapies.

Regarding the second point, progress should be more rapid given that the transformation of F motoneurons towards the S phenotype can be accomplished with chronic electrical stimulation and to a lesser extent exercise [40], [41]. Although chronic electrical stimulation has not yet been studied in an ALS model, different exercise regimes have been shown to modestly extend life in fALS^G93A^ mice [42], [43], [44]. It remains to be determined to what extent, if any, the conversion of motoneurons to more resilient subtypes (e.g. S>FF_fi,fr_>F_ff_) may have contributed to this exercise-induced protection. Clinical investigations of varying exercise regimes in ALS patients have been shown to both lessen disease symptoms and improved function [45], [46], [47], nevertheless there remains some concern among clinicians about advocating exercise to ALS patients because of the conflicting epidemiological reports on whether physical activity may [48], [49], [50], [51] or may not [52], [53], [54] be a disease risk factor.

## Materials and Methods

### Rats

Experiments were performed on fALS^G93A^ rats, which were transgenically engineered to express the human Cu^+2^/Zn^+2^ superoxide dismutase 1 gene with a glycine to alanine base pair mutation at its 93^rd^ codon [55]. The founders for the fALS^G93A^ colony were obtained from Taconic Farms (Germantown, NY) and bred locally. Genotyping was performed by PCR as previously described [56]. All procedures were approved by the Institutional Animal Care and Use Committee of Emory University.

### Surgery

All surgeries were performed on 10 wk old fALS^G93A^ rats. While anesthetized with 2% isoflurane, an incision was made parallel to and below the femur, and the tibial nerve was exposed. In the Crush group (n = 9), the tibial nerve was crushed with jeweler's forceps for 10 sec at the level of the knee. The crush site was visually inspected to confirm that all axons were severed as indicated by a translucent appearance. In the Sham group (n = 9), following a 10 sec exposure of the tibial nerve, the wound was closed with the nerve intact. To control for gender or generational differences the Crush and Sham groups were balanced with littermates of both sexes.

### Behavioral Analysis

Baseline motor behavioral tests were performed by a blinded tester 1–3 days prior to surgery, and then post-operatively 1 x/wk until end-stage, which was defined as the day at which they were unable to right themselves within 30 sec of being placed on either side [55]. Motor behavioral measurements included hindlimb grip strength (GS), Basso-Beatti-Bresnahan (BBB) locomotor rating [57], and ALS motor score [11]. The GS measurements were made with an automated GS meter (Columbus Instruments, Columbus, OH) with the average of 3–6 trials recorded. Disease onset was defined retrospectively as the day when rats reached their maximum body weight.

### Neuropathology

Post-operatively fALS^G93A^ rats were perfused with 4% paraformaldehyde (PFA) at end-stage. To serve as pre-operative controls several fALS^G93A^ rats were perfused at age 10 wk (n = 4). Tissues were processed for assessment of motoneurons, ventral root (VR) axons, and NMJs.

### Motoneurons

A segment of the spinal cord was isolated from L3-S1. It was cryoprotected in 30% sucrose until sinking, immersed in OCT, flash frozen in dry ice cooled isopentane, and cryostat sectioned on to slides at 20 µm. The tissue was air dried overnight, stained with cresyl violet, and mounted with Permount (SP15-100, Fisher Scientific, Fair Lawn, NJ). Every 18th section (i.e. 360 µm interval; 6–8 sections/rat) was imaged from the L4–6 spinal cord. Images were acquired at 20X magnification using a Nikon DS-Fi1 color digital camera on a Nikon E400 microscope and analyzed using the NIS-Elements software (Nikon Instruments Inc, Melville, NY). All Nissl-stained neurons in the ventral horn that had a distinct nucleus were manually traced to determine area. Conservatively, ventral horn neurons with areas ≥300 µm were counted as motoneurons [56].

### Ventral Roots

A 3–4 mm segment of the right L4 VR was dissected from the spinal cord, post-fixed in 4% PFA, treated with 1% tetroxide for 90 min, dehydrated through graded alcohols, and embedded in Epon plastic (EM Sciences, Cincinnati, OH). Cross-sections (720 nm) were stained with toluidine blue, rinsed, and mounted with Permount (Fisher Scientific). Images were acquired as described above for motoneurons except the magnification used was 100X. Myelinated axons were measured using NIS-element software.

### Neuromuscular Junctions

The medial gastrocnemius (MG) muscle was isolated from the right leg, immersed in 20% sucrose mixed with OCT (1∶2), flash frozen in dry ice-cooled isopentane, and the mid-belly of its proximal compartment was cryostat sectioned on to slides at 20 µm. The tissue was air dried overnight, blocked for 1 hr at room temperature in PBS containing 0.3% triton x-100 and 2.5% bovine serum albumin (BSA), incubated overnight at 4°C in rabbit anti-neuronal class III β-tubulin (1∶1000; PRB-435P, Covance, Emeryville, CA) and rabbit anti-synaptophysin (1∶100; Invitrogen, Carlsbad, CA) diluted in blocking solution, washed several times with PBS, incubated overnight at 4°C in goat anti rabbit IgG secondary antibody conjugated to Alexa Fluoro 488 (1∶500; Invitrogen) and rhodamine conjugated α-bungarotoxin (1∶100; Invitrogen) diluted in blocking solution, washed several times in PBS, and then mounted in glycerol-PBS (1∶1) mixture. Images were acquired as described above for motoneurons. The percentage of innervated NMJs was determined based on the co-localization of the pre-synaptic (class III β-tubulin and synaptophysin) and post-synaptic (α-bungarotoxin) markers.

### Statistics

Mean values (± standard error of the mean; SE) are shown throughout. The student's *t*-test was used to make comparisons between time-matched Crush and Sham data. The Kaplan-Meier curves were generated with Sigmaplot software (Systat Software, Chicago, IL). One-way ANOVAs were used to make comparisons for the motoneuron and NMJ quantifications. If the F-critical value was exceeded, Tukey's Honestly Significant Difference post hoc test was then applied.
